# GSNOR facilitates antiviral innate immunity by restricting TBK1 cysteine *S*-nitrosation

**DOI:** 10.1016/j.redox.2021.102172

**Published:** 2021-10-18

**Authors:** Qianjin Liu, Tianle Gu, Ling-Yan Su, Lijin Jiao, Xinhua Qiao, Min Xu, Ting Xie, Lu-Xiu Yang, Dandan Yu, Ling Xu, Chang Chen, Yong-Gang Yao

**Affiliations:** a Key Laboratory of Animal Models and Human Disease Mechanisms of the Chinese Academy of Sciences & Yunnan Province, Kunming Institute of Zoology, Kunming, Yunnan, 650204, China; b Kunming College of Life Science, University of Chinese Academy of Sciences, Kunming, Yunnan, 650204, China; c National Laboratory of Biomacromolecules, Chinese Academy of Sciences Center for Excellence in Biomacromolecules, Institute of Biophysics, Beijing, 100101, China; d KIZ-CUHK Joint Laboratory of Bioresources and Molecular Research in Common Diseases, Kunming Institute of Zoology, Chinese Academy of Sciences, Kunming, Yunnan, 650204, China; e CAS Center for Excellence in Brain Science and Intelligence Technology, Chinese Academy of Sciences, Shanghai, 200031, China

**Keywords:** GSNOR, *S*-nitrosation, Innate immunity, TBK1, Antiviral response

## Abstract

Innate immunity is the first line of host defense against pathogens. This process is modulated by multiple antiviral protein modifications, such as phosphorylation and ubiquitination. Here, we showed that cellular *S*-nitrosoglutathione reductase (GSNOR) is actively involved in innate immunity activation. GSNOR deficiency in mouse embryo fibroblasts (MEFs) and RAW264.7 macrophages reduced the antiviral innate immune response and facilitated herpes simplex virus-1 (HSV-1) and vesicular stomatitis virus (VSV) replication. Concordantly, HSV-1 infection in *Gsnor*^−/-^ mice and wild-type mice with GSNOR being inhibited by N6022 resulted in higher mortality relative to the respective controls, together with severe infiltration of immune cells in the lungs. Mechanistically, GSNOR deficiency enhanced cellular TANK-binding kinase 1 (TBK1) protein *S*-nitrosation at the Cys423 site and inhibited TBK1 kinase activity, resulting in reduced interferon production for antiviral responses. Our study indicated that GSNOR is a critical regulator of antiviral responses and *S*-nitrosation is actively involved in innate immunity.

## Highlights

• GSNOR deficiency impairs antiviral innate response.
• GSNOR deficiency enhances *S*-nitrosation of TBK1 on Cys423 inhibits TBK1 activity.
• Deficiency of *Gsnor* promotes viral infection *in vivo*.
• Mice treated with GSNOR inhibitor N6022 are permissive to HSV-1 infection.

## 1 Introduction

Innate immunity is a crucial first line of defense in hosts against infections. Innate immunity relies on pattern recognition receptors (PRRs) to recognize pathogen-associated molecular patterns (PAMPs). Retinoic acid-inducible gene I (RIG-I)-like receptors (RLRs, including RIG-I and MDA5) and cyclic guanosine monophosphate-adenosine monophosphate synthase (cGAS) are major intracellular PRRs that recognize RNA and DNA viruses, respectively [1,2]. While RLRs recruit mitochondrial antiviral-signaling protein (MAVS, also known as IPS-1, VISA, or CARDIF) and cGAS utilizes the stimulator of IFN genes (STING; also known as MITA, TMEM173, MPYS, or ERIS) for antiviral signaling transduction, these factors converge on TANK-binding kinase 1 (TBK1) [1,2]. TBK1 is a key kinase involved in interferon regulatory factor 3 (IRF3) activation and is crucial for type I interferon (IFN-1) production [3]. Released IFNs bind to cell-surface receptors and activate the transcription of many interferon-stimulated genes (ISGs), such as MX dynamin-like GTPase 1 (*Mx1*) and interferon-induced protein with tetratricopeptide repeats 1 (*Ifit1*), which are effectors for establishing antiviral responses [4,5]. The activation of innate immunity is strictly regulated to maintain immune homeostasis [[1], [2], [3],^6^].

Protein post-translational modifications (PTMs) are very important for maintaining antiviral immune homeostasis. The best characterized PTMs, i.e., phosphorylation and ubiquitination, as well as other PTMs, such as SUMOylation, methylation, and acetylation, are reported to control antiviral signaling via reversible PTM of virus sensors and downstream signaling molecules [[7], [8], [9], [10], [11]]. *S*-nitrosation is a selective covalent PTM that involves the addition of a nitrosyl group to the reactive thiol group of a cysteine to form *S*-nitrosothiol (SNO), a key mechanism in transferring nitric oxide (NO)-mediated signals [12,13]. Cellular *S*-nitrosation levels are controlled by the balance between *S*-nitrosation and denitrosation. While *S*-nitrosation is a non-enzymatic reaction (except for prokaryotes), denitrosation can be a non-enzymatic or enzymatic reaction [13]. *S*-nitrosoglutathione reductase (GSNOR), a highly evolutionarily conserved enzyme of the denitrosylating enzymatic system, modulates *S*-nitrosation through catabolism of *S*-nitrosoglutathione (GSNO) [14]. Genetic deletion of *Gsnor* in mice can cause excessive protein *S*-nitrosation [[15], [16], [17]]. Impaired GSNOR activity, as indicated by abnormal *S*-nitrosation levels, has been found to be associated with a variety of diseases, including cancer, cardiovascular, aging and neurodegenerative diseases [12,[18], [19], [20], [21], [22], [23]]. GSNOR is also actively involved in adaptive immunity [19], especially in plants [24]. Thus, we speculate that GSNOR-maintained *S*-nitrosation may play an active role in antiviral responses.

In this study, based on GSNOR knockout (KO) mice and cells, we showed that GSNOR is actively involved in antiviral immune responses against viral infection. We revealed the underlying mechanism regarding the role of GSNOR deficiency, which enhanced *S*-nitrosation of TBK1 on Cys423, reduced TBK1 kinase activity, and decreased IFN-I production. Our findings suggest that GSNOR is a key regulator of innate immunity against viral infections and may represent a therapeutic target for antiviral treatment.

## 2 Materials and methods

### 2.1 Antibodies and reagents

The following antibodies were used in this study: rabbit polyclonal anti-S-nitrosylated-cysteine (Alpha Diagnostic International; NISC11-A; 1:1000), rabbit polyclonal anti-GSNOR (Abcam; ab175406; 1:1000), rabbit monoclonal anti-IRF3 (Cell Signal Technology; 4302; 1:1000), rabbit monoclonal anti-phospho-IRF3 (Ser396) (Cell Signal Technology; 4947; 1:1000), rabbit monoclonal anti-TBK1 (Cell Signal Technology; 38066; 1:1000), rabbit monoclonal anti-phospho-TBK1 (Ser172) (Cell Signal Technology; 5483; 1:1000), mouse monoclonal anti-Flag (Abmart; TT0003; 1:1000), mouse monoclonal anti-Myc (Invitrogen; R950-25; 1:1000), mouse monoclonal anti-His (Abmart; M20001S; 1:1000), rabbit IgG isotype control (Invitrogen; 31235; 1:500), mouse monoclonal anti β-actin (Beijing Zhong Shan-Golden Bridge Biological Technology Co., Ltd.; TA-09; 1:10000), mouse monoclonal anti-tubulin (EnoGene; E1C601; 1:10000), Alexa Fluor® 488-conjugated anti-rabbit IgG (Invitrogen, A-11008; 1:500), peroxidase-conjugated anti-mouse antibody (KPL; 474–1806; 1:10000), and peroxidase-conjugated anti-rabbit antibody (KPL; 474–1516; 1:10000). The following reagents were used in this study: S-nitrosoglutathione (GSNO) (Santa Cruz; sc-200349A), glutathione (GSH) (Selleckchem; S4606), N6022 (TargetMol; T6901), high molecular weight poly I:C (poly I:C) (InvivoGen; Tlrl-pic), and HSV-60 naked (InvivoGen; Tlrl-hsv60n).

### 2.2 Cell culture

Human keratinocytes (HaCaT cell line), mouse fibroblasts (L929 cell line), Vero cells and monocytes/macrophages (RAW264.7 cell line) were supplied by the Kunming Cell Bank, Kunming Institute of Zoology, Chinese Academy of Sciences. The HaCaT and Vero cells were cultured in Dulbecco's Modified Eagle Medium (DMEM; Gibco). The RAW264.7 and L929 cells were cultured in RPMI 1640 medium (Gibco). All culture media were supplemented with 10% fetal bovine serum (FBS; Gibco, 10099–141) and 1 × penicillin/streptomycin (Gibco, 15140122) at 37 °C in 5% CO_2_.

Mouse peritoneal macrophages (PMs) were isolated from peritoneal cavities 4 days after 3% sodium thioglycolate intraperitoneal injection. Cells were cultured in RPMI 1640 medium with 10% FBS (Gibco, 10099–141) [25]. Mouse embryo fibroblasts (MEFs) were harvested following standard protocols [26]. Briefly, mouse embryos at days 13 and 14 (E13–14) were minced into pieces and trypsinized for 10 min at 37 °C, with the reaction then terminated by adding MEF medium (DMEM with 20% FBS). Cells were washed using fresh MEF medium, then added to a T75 cell culture flask for growth. Cells at passages 2–5 were used for experiments.

### 2.3 Plasmids and transfection

Expression vectors for GSNOR, TBK1, and IRF3 truncate (IRF3-5D [27,28]) were purchased from the Miaoling Plasmid Sharing Platform (MLPSP). Site-directed mutants of GSNOR (p.R115D) and TBK1 (p.C423S, p.C426S, and p.C471S) were constructed using the Easy Mutagenesis System (Beijing TransGen Biotech) according to the manufacturer's protocols. All constructs were confirmed by DNA sequencing. The transient transfection of vectors was performed using Lipofectamine 3000 (Invitrogen, L3000015).

### 2.4 Virus infections

Type 1 herpes simplex virus (HSV-1), vesicular stomatitis virus (tagged by GFP; VSV-GFP), and Sendai virus (SeV) have been described in our previous study [29]. Cells were seeded in 24-well plates (5 × 10^4^ cells/well) for 12 h and washed three times with phosphate-buffered saline (PBS) before infection with HSV-1 (multiplicity of infection [MOI] = 1), VSV-GFP (MOI = 0.01), or SeV [20 hemagglutinating units [HAU]/mL] for 1 h in DMEM without FBS. Infected cells were rinsed and cultured in fresh growth medium containing 1% FBS until harvest. The VSV replication within cells was quantified using flow cytometry.

The plaque assays for HSV-1 infection were performed as described in our previous study [30]. Briefly, the HSV-1 infected cell supernatants at 48 h post-infection were cleared of cell debris by centrifugation at 600*g* for 5 min at room temperature, and serially diluted with DMEM. The Vero cells were seeded in a 6-well plate and incubated with the supernatant dilutions in duplicate or triplicate for 1 h in 37 °C. Cells were washed with DMEM three times, overlaid with a mixture of 2 × DMEM supplemented with FBS and 2% agarose (vol/vol = 1:1), and incubated at 37 °C until the plaque formation was observed, the cells were fixed by covering the agarose layer with 4% paraformaldehyde, followed by staining with 0.2% crystal violet in 50% methanol for 20 min. The dye was washed off and plaques were counted. The VSV replication was quantified by flow cytometry. Briefly, cells were infected with VSV-GFP (MOI = 0.01) for 12 h. Percentage of 10 000 cells expressing GFP (GFP^+^ cells) was counted by flow cytometry.

### 2.5 HSV-1 infection in mouse models

The *Gsnor* KO (*Gsnor*^*−/−*^) mice on a C57BL/6 J background were described in our previous study [31]. All mice were kept in specific pathogen-free conditions within the Experimental Animal Center at the Kunming Institute of Zoology, Chinese Academy of Sciences. All animal experimental procedures and protocols were approved by the Institutional Review Board of the Kunming Institute of Zoology, Chinese Academy of Sciences (approval no: SMKX-20200617-06).

Eight-to ten-week-old *Gsnor*^*−/−*^ mice and WT controls were infected intraperitoneally with HSV-1 (1 × 10^7^ plaque-forming units (pfu)/mouse) as indicated. Mice were monitored daily over time. Mice were euthanized on the indicated days after infection, and lung tissue samples were collected for histological analyses.

### 2.6 CRISPR/Cas9-mediated KO of *Gsnor* in RAW264.7 and L929 cells

CRISPR/Cas9-mediated KO of the *Gsnor* gene in the RAW264.7 and L929 cells was performed according to our previously published procedure [32]. Briefly, small guide RNAs (sgRNAs) (*Gsnor*-sgRNA-forward [F]: CACCGTAAGGCTGCAGTCGCCTGGG/*Gsnor*-sgRNA-reverse: AAACCCCAGGCGACTGCAGCCTTAC) targeting *Gsnor* were annealed and cloned into the pX330-T7 vector expressing mCherry. The RAW264.7 and L929 cells were transfected with the pX330-T7 vector carrying the sgRNAs using Lipofectamine 3000 (Invitrogen, L3000015). The transfected cells expressing mCherry were sorted by flow cytometry and cultured for 48 h. Single cells were manually picked with a mouth pipette for expansion for 3 weeks. The KO of the endogenous GSNOR protein was validated by Western blot analysis.

### 2.7 Quantitative real-time polymerase chain reaction (qRT-PCR) and Western blotting

Total RNA was isolated from the cells using TRIZOL (Invitrogen, 15596–018). Total RNA (1 μg) was used to synthesize single-strand cDNA using M-MLV Reverse Transcriptase (Promega, M170A) in a final volume of 25 μL according to the manufacturer's instructions. The relative mRNA levels of interferon beta 1 (*Ifnb1*), interferon alpha 4 (*Ifna4*), *Mx1*, and *Ifit1* were quantified using qRT-PCR, with normalization to *Actin*. qRT-PCR was performed in a total volume of 20 μL containing 2 μL of diluted products, 10 μL of SYBR Master Mix (Takara), 0.2 μL of 10 μM each primer (Table S1) on a BioRad Real-time PCR detection system. The qRT-PCR thermal cycling conditions included a denaturation cycle at 95 °C for 5 min, followed by 40 cycles of 95 °C for 10 s and 57 °C for 30 s.

Western blot assays for the respective target proteins were performed using standard procedures. Cell lysates of cultured mouse PMs, MEFs, L929, RAW264.7, and HaCaT cells were prepared as described in our previous study [33]. A total of 25 μg of protein was separated by 12% or 15% sodium dodecyl sulphate-polyacrylamide gel electrophoresis (SDS-PAGE) and transferred to a polyvinylidene difluoride membrane (BioRad, L1620177 Rev D). The membrane was soaked with 5% (w/v) skim milk for 2 h at room temperature. Peroxidase-conjugated anti-mouse (lot number 474–1806) or anti-rabbit (lot number 474–1516) IgG (1:5000; KPL) were used as the respective secondary antibodies. The epitope was visualized using an ECL Western Blot Detection kit (Millipore, WBKLS0500). ImageJ (National Institutes of Health, Bethesda, Maryland, USA) was used to evaluate densitometry.

### 2.8 The biotin switch assay

The *S*-nitrosation modification was detected as previously described [15]. Briefly, cells were lysed in HENS buffer. The free cysteine thiols were blocked with methyl methanethiosulfonate (MMTS) at room temperature for 30 min, and excessive MMTS was removed by ice-cold acetone precipitation followed by centrifugation at 2000×*g* for 10 min. The precipitate was resuspended in HENS buffer containing 2.5% SDS with 0.4 mM biotin-maleimide and 10 mM ascorbate and incubated at 37 °C for 1 h or at room temperature for 2 h. The excessive biotin-maleimide was removed by ice-cold acetone precipitation following the above described procedure. The pellet was resuspended in HENS buffer with 200 mM DTT and incubated for 15 min at 100 °C, followed by addition neutralization buffer (250 mM HEPES pH 7.7, 100 mM NaCl, 0.1 mM EDTA, 10 mM neocuproine) and streptavidin-agarose (50–100 μl/sample) to purify biotinylated proteins. The product was subjected to 12% SDS-PAGE.

### 2.9 TBK1 kinase assay

We performed TBK1 kinase assay according to previously published procedure [34]. Briefly, the 293T cells were transfected with expression vector for His-tagged TBK1 (His-TBK1), along with or without other vectors for 24 h, followed by GSNO (500 μM) treatment for 12 h. Cells were harvested and lysed with cell lysis buffer. The whole cell lysate (WCL; 1 mg) was incubated with anti-His magnetic beads (Beyotime, P2135, 30 μL) overnight at 4 °C. Beads were washed twice with cell lysis buffer, followed by two washes with 1 × kinase buffer (Cell Signal Technology, 9802S). The TBK1 kinase assay was performed by incubating washed beads with kinase buffer, 1 mM ATP (Cell Signal Technology, 9804S) and 1 μg recombinant IRF3 as the substrate at 30 °C for 2 h in a total volume of 50 μL. The product was separated on 12% SDS-PAGE and immunoblotted by anti-phospho-IRF3.

### 2.10 Immunofluorescence assay

Cells were fixed with 4% paraformaldehyde for 10 min and incubated with the indicated primary antibody against S-nitrosylated-cysteine (1:500) overnight at 4 °C. After three washes with PBS (5 min each), immunoreactivity was detected using the FITC-conjugated anti-rabbit IgG (1:500; Invitrogen, A-21206) for 1 h at room temperature. Nuclei were counterstained with 1 μg/ml DAPI (10236276001; Roche Diagnostics), and the slides were visualized under a FluoView 1000 confocal microscope (Olympus). IgG (Invitrogen; 31235) staining was used as a control.

### 2.11 Histopathological staining

Lung tissues from mice with or without HSV-1 infection (1 × 10^7^ plaque-forming units (pfu)/mouse) or N6022 (5 mg/kg) treatment were fixed with 10% phosphate-buffered formalin, embedded into paraffin. The embedded tissue was sectioned into 4 μm thick sections, then the sections stained with hematoxylin and eosin (H&E) solution, and examined by microscopy (Leica, Germany) for histological analysis.

### 2.12 Statistical analysis

All statistical analyses were performed using GraphPad Prism v7.0. Student's *t-*test was used for statistical analysis between groups, and the log-rank (Mantel-Cox) test was used for statistical analysis of the survival curves. All tests were two-tailed. All data are represented as mean ± standard deviation (SD). A *P-*value of <0.05 was considered as statistically significant.

## 3 Results

### 3.1 GSNOR deficiency impairs IFN-1 production and promotes viral replication

**Fig. 1:**
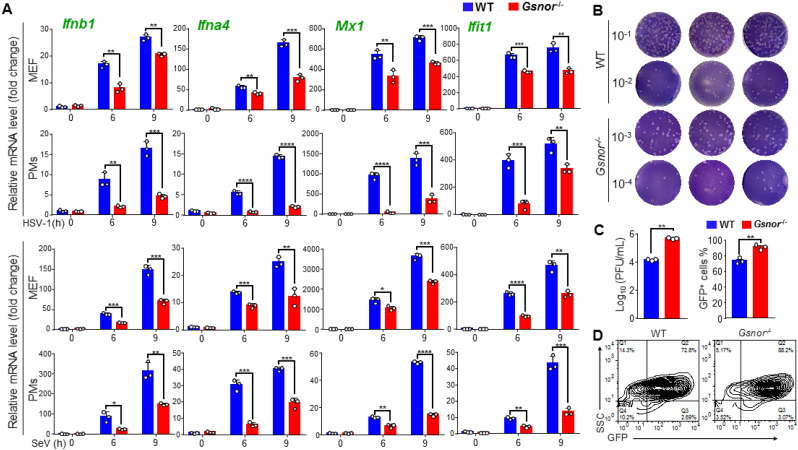
*Gsnor* deficiency impairs type I interferon production and promotes viral replication, **(A)** Up-regulation of mRNA levels of *Ifnb-1*, *Ifna4*, *Mx1*, and *Ifit1* in peritoneal macrophages or MEFs from WT or *Gsnor*^−/−^ mice upon HSV-1 (MOI = 1) or SeV (20 HAU/mL) infection for indicated times. **(B and C)** Enhanced viral replication in MEFs with GSNOR KO relative to WT cells. Viral titers in supernatant of HSV-1-infected MEF cells were analyzed by plaque assay (**B**) and quantified (**C**, *left*). Vero cells were infected with different dilutions of HSV-1 in culture supernatant of HSV-1-infected MEFs (10^−1^ and 10^−2^ for WT MEFs, 10^−3^ and 10^−4^ for GSNOR KO MEFs). **(D)** Measurement of VSV replication in MEF cells with or without GSNOR by flow cytometry. MEF Cells (2 × 10^5^) were infected with VSV-GFP (MOI = 0.01) for 12 h, and percentage of 10 000 cells expressing GFP (GFP^+^ cells) was quantified (**C**, *right*). All data represent three independent experiments with similar results. Data are mean ± SD. *P < 0.05, **P < 0.01, ***P < 0.001, two-tailed unpaired Student's *t*-test.

To test whether GSNOR is involved in antiviral innate immune responses, we subjected MEFs and PMs from wild-type (WT) and GSNOR KO mice to DNA (HSV-1) and RNA viral (Sendai virus, SeV) infection, respectively. GSNOR deficiency in the PMs and MEFs impaired the mRNA expression levels of *Ifnb1* and *Ifna4*, as well as several ISGs, including *Mx1* and *Ifit1* (Fig. 1A). Previous studies have shown that transfected nucleic acid mimics, such as RNA virus mimic poly (I:C) [35] and DNA virus mimic HSV-60 [36], are efficient at inducing the mRNA expression of downstream antiviral cytokines [29,37,38]. We found that GSNOR deficiency inhibited RNA virus mimic poly (I:C) and DNA virus mimic HSV-60-mediated IFN-β and ISG expression (Fig. S1).

We further investigated the antiviral capabilities of GSNOR using plaque assays. GSNOR KO promoted HSV-1 replication in the MEFs (Fig. 1B and **C *left*)**. Similarly, VSV replication also increased in the *Gsnor*^*−/−*^ MEFs relative to the WT cells (Fig. 1D and **C *right***). Collectively, these data suggest that GSNOR is actively involved in antiviral immune responses by positively modulating IFN-1 signaling.

### 3.2 GSNOR is crucial for antiviral innate immune response *in vivo*

**Fig. 2:**
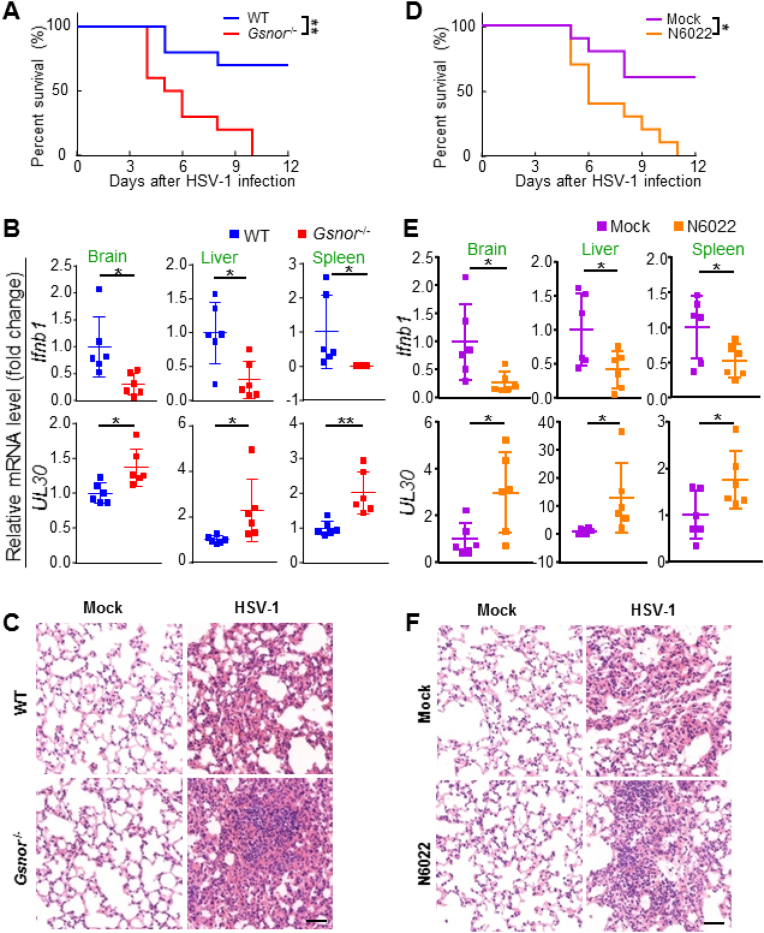
GSNOR deficiency inhibits innate immune responses against HSV-1 infection *in vivo,***(A)** Kaplan-Meier survival curves of different groups of mice (WT and *Gsnor*^*−/−*^ mice) in response to HSV-1 infection (n = 10 mice per group). **(B)***Ifnb1* mRNA expression levels (*upper*) and viral burden of HSV-1 (*below*) in tissues from HSV-1-infected WT or *Gsnor*^−/-^ mice (n = 6 mice per group). Measurement for *Ifnb1* and HSV-1 *UL3*0 mRNA levels in brain, spleen, and liver tissues was performed using qRT-PCR. **(C)** Hematoxylin and eosin staining of lung tissue sections from WT or *Gsnor*^*−/−*^ mice with or without HSV-1 infection. Scale bar, 50 μm. **(D)** Kaplan-Meier survival curves of different groups of mice (WT mice with or without GSNOR inhibition) in response to HSV-1 infection (n = 10 mice per group). **(E)***Ifnb1* mRNA expression level (*upper*) and viral burden of HSV-1 (*below*) in tissues from HSV-1-infected mice with (N6022) or without (mock) pretreatment (n = 6 mice per group). **(F)** Hematoxylin and eosin staining of lung tissue sections from HSV-1-infected mice with (N6022) or without (mock) pretreatment. Scale bar, 50 μm. Statistical significance was determined by log-rank Mantel-Cox test in **(A and D)** and two-tailed unpaired Student's *t-*test in **(B and E)**. Data are mean ± SD in (**B and E)**. *P < 0.05, **P < 0.01.

Next, we investigated the function of GSNOR in antiviral innate immune response *in vivo*. We compared the survival rates between WT and *Gsnor*^*−/−*^ mice after infection with HSV-1. Results showed that *Gsnor*^*−/−*^ mice were more susceptible to HSV-1, with a higher mortality than their WT counterparts (Fig. 2A). Consistently, the *Ifnb1* mRNA level induced by HSV-1 infection was significantly lower in the tissues of *Gsnor*^−/−^ mice than in the tissues of WT mice (Fig. 2B). In agreement with this pattern, a higher viral burden was observed in the spleen, liver, and brain tissues of *Gsnor*^−/−^ mice than in the same tissues of WT mice (Fig. 2B). Histological analyses showed greater infiltration of immune cells and higher level of lung tissue impairment in *Gsnor*^−/−^ mice than in WT mice (Fig. 2C).

We used a specific and potent GSNOR inhibitor (N6022) [39] to further confirm the antiviral role of GSNOR. Results showed that mice injected with N6022 were more susceptible to HSV-1 infection than the control mice (Fig. 2D). *Ifnb1* mRNA expression was also significantly lower in the tissues of N6022-treated mice than in the tissues of control mice after HSV-1 infection (Fig. 2E). Consistently, HSV-1 production was higher in the tissues of N6022-treated mice than in the tissues of the controls (Fig. 2E). Severe infiltration of immune cells was observed in the lungs of N6022-treated mice compared with control mice after HSV-1 infection (Fig. 2F). Collectively, these *in vivo* results indicate that GSNOR is a critical regulator of the antiviral immune response to viruses.

### 3.3 GSNOR enhances innate antiviral immunity through enzymatic activity

**Fig. 3:**
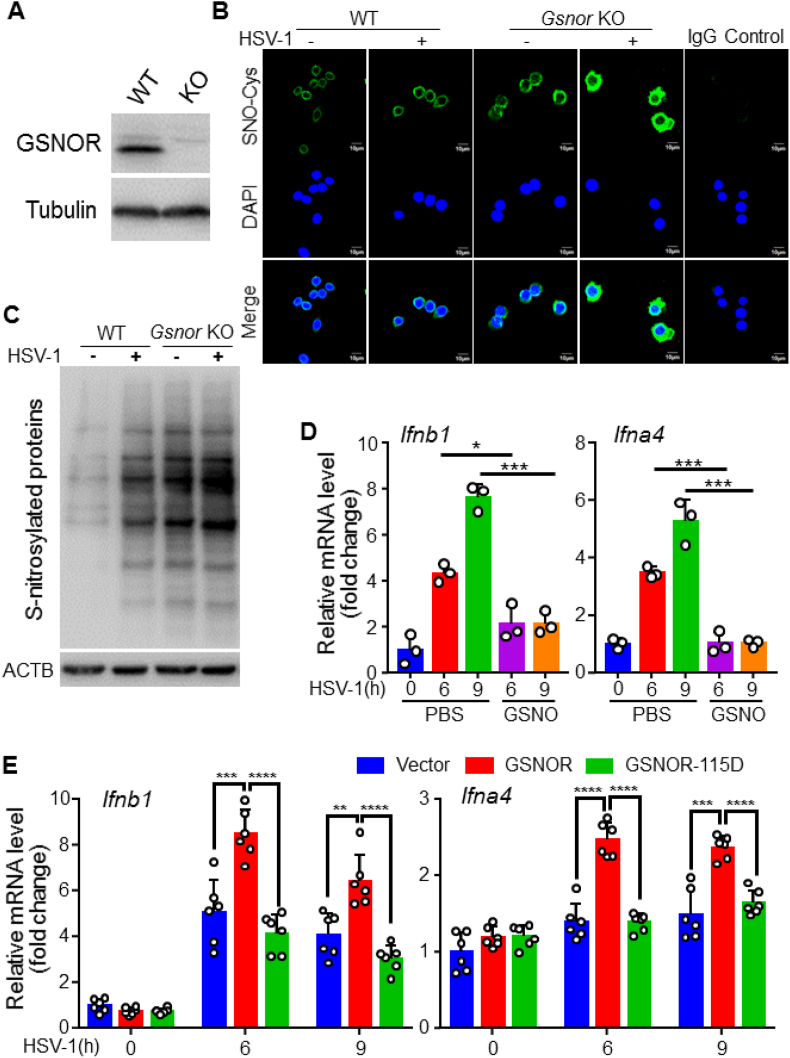
GSNOR positively regulates innate antiviral immunity through enzymatic activity, **(A)** Western blotting of GSNOR expression in WT or GSNOR KO RAW264.7 cells. **(B)** Representative fluorescence imaging of RAW264.7 WT and KO cells stained with anti–SNO–Cys antibody (SNO-Cys, green). DAPI (blue) staining was used to highlight nuclei, IgG staining was used to normal control. **(C)** Western blotting of total SNO-proteins in RAW264.7 WT and KO cells infected with (+) or without (−) HSV-1 for 6 h. **(D)** GSNO pretreatment inhibited induced *Ifnb1* and *Ifna4* mRNA expression by HSV-1 compared to control group with PBS pretreatment. **(E)** Overexpression of GSNOR, but not GSNOR-115D mutant, in *Gsnor* KO RAW264.7 cells promoted *Ifnb1* and *Ifna4* mRNA expression induced by HSV-1 infection. All data represent three independent experiments with similar results. Data are mean ± SD. *P < 0.05, **P < 0.01, ***P < 0.001, ****P < 0.0001, two-tailed unpaired Student's *t*-test. (For interpretation of the references to colour in this figure legend, the reader is referred to the Web version of this article.)

To test whether GSNOR regulates antiviral innate immunity through enzymatic activity, we constructed GSNOR knockout Raw264.7 cells (Fig. 3A) and detected protein *S*-nitrosation (SNO-protein) in WT and *Gsnor* KO RAW264.7 cells with or without HSV-1 infection. Based on an immunofluorescent assay with anti–SNO–Cys antibody staining, we found a remarkable increase of the level of SNO-protein in the *Gsnor* KO RAW264.7 cells compared to WT cells (Fig. 3B). Consistently, HSV-1 infection resulted in a significant increase of the level of SNO-protein in the *Gsnor* KO RAW264.7 cells compared with the WT cells (Fig. 3C). These results indicated that HSV-1 infection increases the amount of *S*-nitrosation on proteins, and GSNOR can reduce the level of SNO-protein induced by viral infection.

GSNO is the substrate of GSNOR and acts as a major endogenous NO donor [14]. Treatment with GSNO can induce SNO-protein formation in cells [40]. To further confirm the involvement of GSNOR in antiviral responses, we incubated RAW264.7 cell with GSNO, followed by HSV-1 infection. We found that GSNO (200 μM) pretreatment significantly inhibited HSV-1 infection-induced *Ifnb1* and *Ifna4* mRNA expression (Fig. 3D), suggesting inhibition of the antiviral response.

To further verify the active role of GSNOR in antiviral signaling, we transiently transfected *Gsnor* KO RAW264.7 cells with Flag-tagged GSNOR and mutant GSNOR-115D (R115D mutant of GSNOR) expression vectors, which inactivate GSNOR enzymatic activity [27,28]. We found that overexpression of WT GSNOR improved *Ifnb1* and *Ifna4* mRNA levels after HSV-1 infection compared with empty vector, whereas overexpression of GSNOR-115D did not (Fig. 3E). Taken together, these results suggest that GSNOR positively regulates innate antiviral immunity, and this capacity is dependent on its enzymatic activity.

### 3.4 GSNOR deficiency potentiates viral replication by inhibiting TBK1-IRF3 signaling

**Fig. 4:**
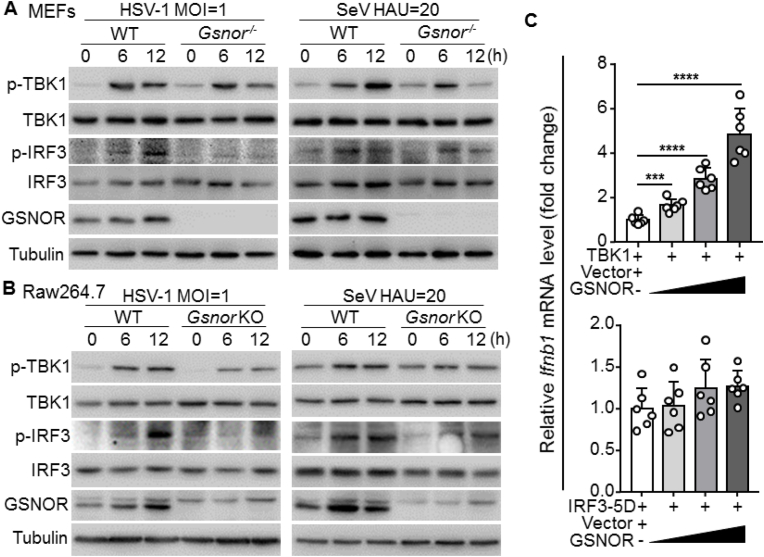
*Gsnor* deficiency potentiates viral replication by inhibiting TBK1-IRF3 signaling, **(A)** Immunoblot of MEF lysates from WT and *Gsnor*^−/−^ mice infected with HSV-1 (MOI = 1) or SeV (20 HAU/mL) for indicated times. GSNOR deficiency reduced phosphorylation levels of IRF3 and TBK1. **(B)** Immunoblots of lysates of WT and GSNOR KO RAW264.7 cells infected with HSV-1 (MOI = 1) or SeV (20 HAU/mL) for indicated times. **(C)** Overexpression of GSNOR and TBK1, but not GSNOR and IRF3-5D mutant, in RAW264.7 cells enhanced *Ifnb1* mRNA expression in a dose-dependent manner. RAW264.7 cells were transfected with combined expression vectors for 24 h, then harvested for quantification of *Ifnb1* mRNA level. All data represent three independent experiments with similar results. Data are mean ± SD. *P < 0.05, **P < 0.01, two-tailed unpaired Student's *t*-test.

Next, we determined the molecular mechanism of GSNOR in the virus-triggered signaling pathway. We explored the hallmarks of the antiviral innate immune response, i.e., phosphorylation of TBK1 and IRF3 (p-TBK1 and p-IRF3, respectively) in response to viral stimulation [41,42]. Consistent with previous observation [43], we found that p-TBK1 and p-IRF3 levels were induced by HSV-1 and SeV infections in the WT MEFs. However, these increases were reduced in the *Gsnor*^*−/−*^ MEFs (Fig. 4A). The phosphorylated TBK1 was decreased at 12 h, which may be caused by negative regulation of the IFN signaling to prevent its hyperactivation and be detrimental to the host [[44], [45], [46]]. We also determined the response of *Gsnor* KO RAW264.7 cells to viral infection. Results showed that the levels of p-TBK1 and p-IRF3 were reduced in GSNOR-deficient cells compared to WT cells after viral infection (Fig. 4B).

We further validated the role of GSNOR in viral infection in human HaCaT cells, which are susceptible to both DNA and RNA virus infections. Consistent with the above results in mouse cells (Fig. 4A and B), GSNOR knockdown using siRNA significantly reduced the levels of p-TBK1 and p-IRF3 upon HSV-1 and SeV infection (Fig. S2), whereas overexpression of GSNOR potentiated virus-induced p-TBK1 and p-IRF3 levels in HaCaT cells compared to the control group (Fig. S3). These results suggested that GSNOR can promote IFN-β expression and antiviral immune response induced by RNA and DNA viruses.

Major cytoplasmic sensors of RNA and DNA viruses, such as RLRs and cGAS, transduce antiviral signaling via TBK1-IFN-β signaling [[1], [2], [3]]. Because GSNOR deficiency impaired IFN expression in response to RNA and DNA virus infections as shown above (Fig. 1), we speculated that GSNOR may affect IFN-I production via targeting TBK1. We examined the effects of GSNOR on the transcription of *Ifnb1* mediated by the overexpression of components of the virus-triggered pathway. We found that GSNOR overexpression activated the TBK1-triggered transcription of *Ifnb1* in a dose-dependent manner but had no effect on IRF3-5D-induced *Ifnb1* mRNA expression (Fig. 4C). Collectively, these data suggest that GSNOR regulates antiviral signaling via TBK1.

### 3.5 *S*-nitrosation of TBK1 on Cys423 inhibits its kinase activity

**Fig. 5:**
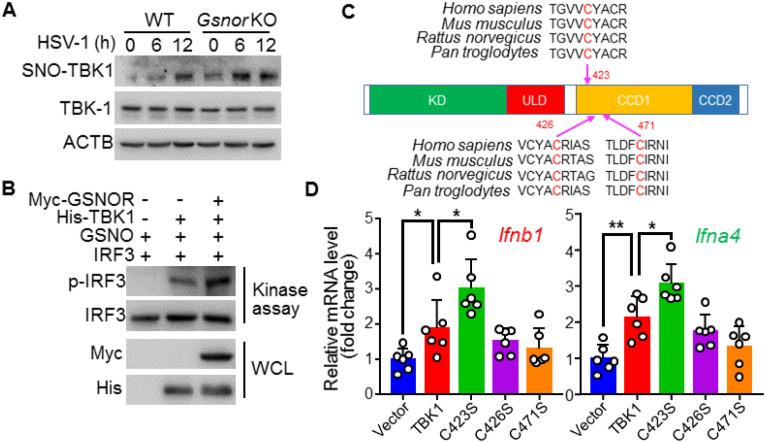
*S*-nitrosation of TBK1 Cys423 inhibits its kinase activity, **(A)** HSV-1 infection induced *S*-nitrosation of TBK1 protein, and this effect was enhanced in *Gsnor* KO L929 cells. **(B)***In vitro* kinase assay of phosphorylated IRF3 and total IRF3 in 293T cells transfected with expression vector His-tagged TBK1, with or without expression vector of GSNOR. (**C)** Diagram of predicted mouse TBK1 domain structure and *S*-nitrosation sites Cys423, Cys426, and Cys471. *S*-nitrosation sites were predicted by iSNO-PseAAC predictor [47] and evolutionary conservation status of these predicted sites was evaluated by comparing TBK1 protein sequences of human, chimpanzee, rat, and mouse. **(D)** Effects of overexpression of WT TBK1 and its mutants at the predicted *S*-nitrosation sites (Cys to Ser). TBK1 mutant C423S enhanced *Ifnb1* and *Ifna4* mRNA levels compared to WT TBK1 and other mutants. All data represent three independent experiments with similar results. Data are mean ± SD. *P < 0.05, **P < 0.01, two-tailed unpaired Student's *t*-test.

To determine why GSNOR regulates innate immunity via TBK1 activity, we first examined the effects of viral infection on TBK1 *S*-nitrosation in WT and *Gsnor* KO L929 cells. We found that viral infection induced *S*-nitrosation of TBK1 in WT cells and the degree of TBK1 *S*-nitrosation was further increased by GSNOR KO (Fig. 5A). Considering the fact that the kinase activity of TBK1 is indispensable for IRF3 activation [34], we performed an *in vitro* kinase assay using recombinant IRF3 to determine whether GSNOR affects the kinase activity of TBK1. We found that GSNOR promotes TBK1 kinase activity and TBK1-mediated phosphorylation of IRF3 (Fig. 5B).

Next we determined potential *S*-nitrosation sites of TBK1. We used an *in silico* prediction program, the iSNO-PseAAC predictor [47], and predicted five Cysteines (Cys89, Cys423, Cys426, Cys471 and Cys558) of TBK1 as potential SNO-modified positions. Among these five positions, three (Cys423, Cys426 and Cys471) are evolutionarily conserved according to a comparison of TBK1 protein sequences of human, chimpanzee, rat and mouse (Fig. 5C). We speculated these conserved Cysteines may be the SNO-sites that mediated the antiviral signaling. We constructed TBK1 expression vectors with cysteine (Cys) mutations at positions Cys423, Cys426, Cys471 to serine. Transfection of these TBK1 mutants into L929 cells was performed in the presence of GSNO. Cells transfected with the Cys423 mutant (p.C423S), but not p.C426S or p.C471S, showed increased IFN expression compared to cells overexpressing WT TBK1 (Fig. 5D). These data indicate that Cys423 of TBK1 is the key *S*-nitrosation site responsible for the effects of TBK1 *S*-nitrosation during antiviral response.

## 4 Discussion

**Fig. 6:**
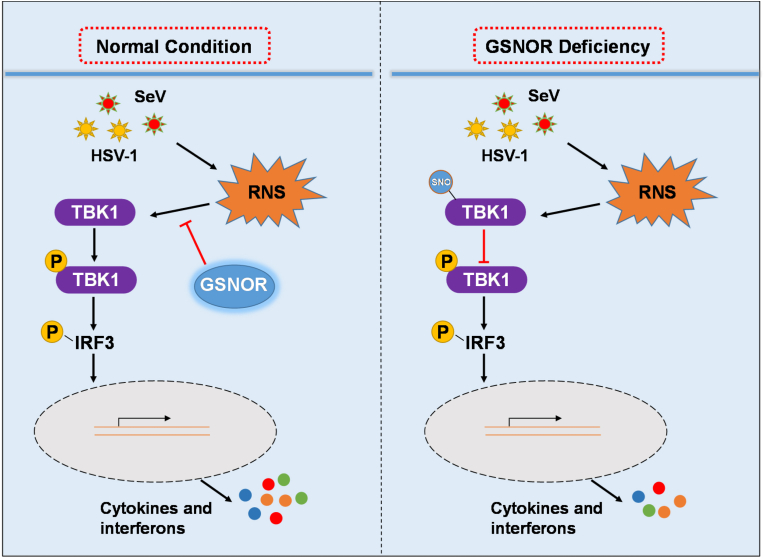
A proposed mechanism of GSNOR in antiviral immune response, GSNOR facilitates antiviral innate immunity by regulating TBK1 *S*-nitrosation, which is impacted by GSNOR deficiency. Virus infection induces reactive nitrogen species (RNS), which contributes to TBK1 *S*-nitrosation and affects downstream innate immune response.

Most physiological processes only operate under a narrow range of conditions and are maintained by specialized homeostatic mechanisms [48,49]. Antiviral immunity balances the elimination of invading pathogens and avoidance of detrimental inflammatory responses in the host, partially via protein modifications [50,51]. As a major cellular denitrosylase, GSNOR reduces the cellular levels of SNO-protein by regulating GSNO and SNO proteins [14]. In mammals, dysregulation of GSNOR contributes to the pathogenesis of a diverse array of chronic diseases [19,20,52,53]. However, the role of GSNOR in antiviral innate immunity is indistinct. In this study, we investigated crosstalk between the antiviral innate immune response and *S*-nitrosation by using cellular models, GSNOR KO mice, and GSNOR inhibitor (N6022)-treated mice. We found that GSNOR deficiency and GSNO pretreatment reduced innate immune antiviral activity, which promoted TBK1 *S*-nitrosation and inhibited its activity, leading to enhanced viral replication. Overexpression of GSNOR facilitated the expression of *Ifnb1* and ISGs, indicating enhanced activation of antiviral signaling. Similarly, overexpression of the GSNOR 115D mutant in GSNOR KO cells could not rescue the deficiency of innate immunity caused by GSNOR KO, suggesting a protective role of GSNOR in antiviral innate immunity via regulation of *S*-nitrosation. In particular, the *in vivo* assays based on *Gsnor*^*–/–*^and N6022-pretreated mice indicated that inhibition or deficiency of GSNOR conferred susceptibility to viral infection. All these results showed that GSNOR strengthened the antiviral innate immune response by preserving TBK1 activity in response to viral infection (Fig. 6).

As a key kinase involved in the IFN regulatory factor 3 (IRF3) activation and IFN-1 production, the expression and phosphorylation levels of TBK1 are precisely modulated by diverse regulators through PTMs to maintain proper activation of innate immunity, thereby efficiently clearing the virus but avoiding IFN-driven autoinflammation [3]. For example, protein phosphatases modulate TBK1 kinase activity through phosphorylation and dephosphorylation [54,55]. The K63-linked polyubiquitination of TBK1 by the E3 ubiquitin ligase TRAF (tumor necrosis factor receptor-associated factor) enhances TBK1 activity and promotes IFN-1 signaling [56], while the E3 ubiquitin ligase DTX4 mediates the K48-linked polyubiquitination of Lys670 and degradation of TBK1 and suppresses IFN production [45]. In addition to ubiquitination and phosphorylation, TBK1 also undergoes other PTMs, such as SUMOylation and deacetylation. SUMOylated K694 affects TBK1 activity [57]. Deacetylation of Lys241 by HDAC9 is critical for TBK1 kinase activity, while ADP-ribosylation by TIPARP suppresses its activity [58,59]. We found that viral infection induced TBK1 *S*-nitrosation at Cys423. This PTM of TBK1 suppressed its activity during the antiviral innate immune response, whereas denitrosation of TBK1 by GSNOR restored its activity. Consistent with these results, mutation of this *S*-nitrosation site (Cys423Ser) of TBK1 enhanced its antiviral capability. These findings extend the PTM of TBK1 and reveal a previously unknown mechanism of antiviral innate immune response regulation by targeting TBK1.

This study has several limitations. First, although we observed an antiviral role of GSNOR, it remains unknown whether GSNOR regulates TBK1-mediated innate immune responses in a macrophage-specific manner, or with other specific immune cell types. Studies with conditional *Gsnor* knockout mice specifically in the macrophages may help to answer this question. Second, as GSNOR is also a formaldehyde dehydrogenase [60], whether formaldehyde metabolism plays a role on the antiviral response needs to be determined. Third, we evaluated the potential SNO-protein sites in TBK1 using an *in silico* prediction, and only tested the potential effects of three evolutionarily conserved sites. It is ideal to run mass spectrometry to identify the specific site, as described in a previous study [61].

In summary, we discovered a link between *S*-nitrosation and antiviral innate immunity and found that GSNOR enhances antiviral innate immunity by denitrosation of TBK1. These findings illustrate a previously unknown strategy of GSNOR in the regulation of antiviral innate immunity, thereby providing a promising therapeutic target for the control of viral infections and IFN-1-associated autoimmune diseases.

## Author contributions

Yong-Gang Yao and Qianjin Liu conceived and designed the experiments. Qianjin Liu, Tianle Gu, Ling-Yan Su, Lijin Jiao, Xinhua Qiao, Min Xu, Ting Xie, Lu-Xiu Yang, Dandan Yu, and Ling Xu performed the experiments and analyzed the data. Chang Chen contributed to experimental design, interpretation of the results, manuscript revision and sharing *Gsnor*^−/−^ mice. Yong-Gang Yao, Qianjin Liu, Tianle Gu, and Ling-Yan Su wrote the manuscript. All authors reviewed the content and approved the final version for publication.

## Declaration of competing interest

There were no potential conflicts of interest to be disclosed.

## References

[1] Ablasser A., Hur S. (2020). Regulation of cGAS- and RLR-mediated immunity to nucleic acids. Nat. Immunol..

[2] Roers A., Hiller B., Hornung V. (2016). Recognition of endogenous nucleic acids by the innate immune system. Immunity.

[3] Zhou R., Zhang Q., Xu P. (2020). TBK1, a central kinase in innate immune sensing of nucleic acids and beyond. Acta Biochim. Biophys. Sin..

[4] Schneider W.M., Chevillotte M.D., Rice C.M. (2014). Interferon-stimulated genes: a complex web of host defenses. Annu. Rev. Immunol..

[5] Wang W., Xu L., Su J., Peppelenbosch M.P., Pan Q. (2017). Transcriptional regulation of antiviral interferon-stimulated genes. Trends Microbiol..

[6] Zhang K., Zhang Y., Xue J., Meng Q., Liu H., Bi C., Li C., Hu L., Yu H., Xiong T., Yang Y., Cui S., Bu Z., He X., Li J., Huang L., Weng C. (2019). DDX19 inhibits Type I interferon production by disrupting TBK1-IKKepsilon-IRF3 interactions and promoting TBK1 and IKKepsilon degradation. Cell Rep..

[7] Mowen K.A., David M. (2014). Unconventional post-translational modifications in immunological signaling. Nat. Immunol..

[8] Zhou Y., He C., Wang L., Ge B. (2017). Post-translational regulation of antiviral innate signaling. Eur. J. Immunol..

[9] El-Asmi F., McManus F.P., Thibault P., Chelbi-Alix M.K. (2020). Interferon, restriction factors and SUMO pathways. Cytokine Growth Factor Rev..

[10] Zheng Y., Gao C. (2020). Fine-tuning of antiviral innate immunity by ubiquitination. Adv. Immunol..

[11] Fiil B.K., Gyrd-Hansen M. (2021). The Met1-linked ubiquitin machinery in inflammation and infection. Cell Death Differ..

[12] Nakamura T., Oh C.K., Zhang X., Lipton S.A. (2021). Protein S-nitrosylation and oxidation contribute to protein misfolding in neurodegeneration. Free Radic. Biol. Med..

[13] Stomberski C.T., Hess D.T., Stamler J.S. (2019). Protein S-nitrosylation: determinants of specificity and enzymatic regulation of S-nitrosothiol-based signaling. Antioxidants Redox Signal..

[14] Liu L., Hausladen A., Zeng M., Que L., Heitman J., Stamler J.S. (2001). A metabolic enzyme for S-nitrosothiol conserved from bacteria to humans. Nature.

[15] Yi W., Zhang Y., Liu B., Zhou Y., Liao D., Qiao X., Gao D., Xie T., Yao Q., Zhang Y., Qiu Y., Huang G., Chen Z., Chen C., Ju Z. (2021). Protein S-nitrosylation regulates proteostasis and viability of hematopoietic stem cell during regeneration. Cell Rep..

[16] Liu L., Yan Y., Zeng M., Zhang J., Hanes M.A., Ahearn G., McMahon T.J., Dickfeld T., Marshall H.E., Que L.G., Stamler J.S. (2004). Essential roles of S-nitrosothiols in vascular homeostasis and endotoxic shock. Cell.

[17] Smith B.C., Marletta M.A. (2012). Mechanisms of S-nitrosothiol formation and selectivity in nitric oxide signaling. Curr. Opin. Chem. Biol..

[18] Tang X., Pan L., Zhao S., Dai F., Chao M., Jiang H., Li X., Lin Z., Huang Z., Meng G., Wang C., Chen C., Liu J., Wang X., Ferro A., Wang H., Chen H., Gao Y., Lu Q., Xie L., Han Y., Ji Y. (2020). SNO-MLP (S-nitrosylation of muscle LIM protein) facilitates myocardial hypertrophy through TLR3 (toll-like receptor 3)-mediated RIP3 (receptor-interacting protein kinase 3) and NLRP3 (NOD-like receptor pyrin domain containing 3) inflammasome activation. Circulation.

[19] Li J., Zhang Y., Zhang Y., Lu S., Miao Y., Yang J., Huang S., Ma X., Han L., Deng J., Fan F., Liu B., Huo Y., Xu Q., Chen C., Wang X., Feng J. (2018). GSNOR modulates hyperhomocysteinemia-induced T cell activation and atherosclerosis by switching Akt S-nitrosylation to phosphorylation. Redox Biol.

[20] Wei W., Li B., Hanes M.A., Kakar S., Chen X., Liu L. (2010). S-nitrosylation from GSNOR deficiency impairs DNA repair and promotes hepatocarcinogenesis. Sci. Transl. Med..

[21] Kartawy M., Khaliulin I., Amal H. (2020). Systems biology reveals reprogramming of the S-nitroso-proteome in the cortical and striatal regions of mice during aging process. Sci. Rep..

[22] Nakamura T., Tu S., Akhtar M.W., Sunico C.R., Okamoto S., Lipton S.A. (2013). Aberrant protein s-nitrosylation in neurodegenerative diseases. Neuron.

[23] Khaliulin I., Kartawy M., Amal H. (2020). Sex differences in biological processes and nitrergic signaling in mouse brain. Biomedicines.

[24] Lubega J., Umbreen S., Loake G.J. (2021). Recent advances in the regulation of plant immunity by S-nitrosylation. J. Exp. Bot..

[25] Zhang X., Goncalves R., Mosser D.M. (2008). The isolation and characterization of murine macrophages. Curr Protoc Immunol Chapter.

[26] Durkin M.E., Qian X., Popescu N.C., Lowy D.R. (2013). Isolation of mouse embryo fibroblasts. Bio Protoc.

[27] Engeland K., Hoog J.O., Holmquist B., Estonius M., Jornvall H., Vallee B.L. (1993). Mutation of Arg-115 of human class III alcohol dehydrogenase: a binding site required for formaldehyde dehydrogenase activity and fatty acid activation. Proc. Natl. Acad. Sci. U. S. A..

[28] Wu K., Ren R., Su W., Wen B., Zhang Y., Yi F., Qiao X., Yuan T., Wang J., Liu L., Izpisua Belmonte J.C., Liu G.H., Chen C. (2014). A novel suppressive effect of alcohol dehydrogenase 5 in neuronal differentiation. J. Biol. Chem..

[29] Xu L., Yu D., Fan Y., Peng L., Wu Y., Yao Y.G. (2016). Loss of RIG-I leads to a functional replacement with MDA5 in the Chinese tree shrew. Proc. Natl. Acad. Sci. U. S. A..

[30] Xu L., Yu D., Peng L., Wu Y., Fan Y., Gu T., Yao Y.L., Zhong J., Chen X., Yao Y.G. (2020). An alternative splicing of *Tupaia* STING modulated anti-RNA virus responses by targeting MDA5-LGP2 and IRF3. J. Immunol..

[31] Zhang Y., Wu K., Su W., Zhang D.F., Wang P., Qiao X., Yao Q., Yuan Z., Yao Y.G., Liu G., Zhang C., Liu L., Chen C. (2017). Increased GSNOR expression during aging impairs cognitive function and decreases S-nitrosation of CaMKIIalpha. J. Neurosci..

[32] Gu T., Yu D., Li Y., Xu L., Yao Y.L., Yao Y.G. (2019). Establishment and characterization of an immortalized renal cell line of the Chinese tree shrew (*Tupaia belangeri chinesis*). Appl. Microbiol. Biotechnol..

[33] Liu Q., Su L.Y., Sun C., Jiao L., Miao Y., Xu M., Luo R., Zuo X., Zhou R., Zheng P., Xiong W., Xue T., Yao Y.G. (2020). Melatonin alleviates morphine analgesic tolerance in mice by decreasing NLRP3 inflammasome activation. Redox Biol.

[34] Wang Y., Wang P., Zhang Y., Xu J., Li Z., Li Z., Zhou Z., Liu L., Cao X. (2020). Decreased expression of the host long-noncoding RNA-GM facilitates viral escape by inhibiting the kinase activity TBK1 via S-glutathionylation. Immunity.

[35] Shen Y., Tang K., Chen D., Hong M., Sun F., Wang S., Ke Y., Wu T., Sun R., Qian J., Du Y. (2021). Riok3 inhibits the antiviral immune response by facilitating TRIM40-mediated RIG-I and MDA5 degradation. Cell Rep..

[36] Sprenger H.G., MacVicar T., Bahat A., Fiedler K.U., Hermans S., Ehrentraut D., Ried K., Milenkovic D., Bonekamp N., Larsson N.G., Nolte H., Giavalisco P., Langer T. (2021). Cellular pyrimidine imbalance triggers mitochondrial DNA-dependent innate immunity. Nat Metab.

[37] Lian H., Zang R., Wei J., Ye W., Hu M.M., Chen Y.D., Zhang X.N., Guo Y., Lei C.Q., Yang Q., Luo W.W., Li S., Shu H.B. (2018). The Zinc-Finger protein ZCCHC3 binds RNA and facilitates viral RNA sensing and activation of the RIG-I-like receptors. Immunity.

[38] Luo W.W., Li S., Li C., Lian H., Yang Q., Zhong B., Shu H.B. (2016). iRhom2 is essential for innate immunity to DNA viruses by mediating trafficking and stability of the adaptor STING. Nat. Immunol..

[39] Green L.S., Chun L.E., Patton A.K., Sun X., Rosenthal G.J., Richards J.P. (2012). Mechanism of inhibition for N6022, a first-in-class drug targeting S-nitrosoglutathione reductase. Biochemistry.

[40] Romero J.M., Bizzozero O.A. (2009). Intracellular glutathione mediates the denitrosylation of protein nitrosothiols in the rat spinal cord. J. Neurosci. Res..

[41] Du M., Liu J., Chen X., Xie Y., Yuan C., Xiang Y., Sun B., Lan K., Chen M., James S.J., Zhang Y., Zhong J., Xiao H. (2015). Casein kinase II controls TBK1/IRF3 activation in IFN response against viral infection. J. Immunol..

[42] Honda K., Takaoka A., Taniguchi T. (2006). Type I interferon gene induction by the interferon regulatory factor family of transcription factors. Immunity.

[43] Meng F., Zhou R., Wu S., Zhang Q., Jin Q., Zhou Y., Plouffe S.W., Liu S., Song H., Xia Z., Zhao B., Ye S., Feng X.H., Guan K.L., Zou J., Xu P. (2016). Mst1 shuts off cytosolic antiviral defense through IRF3 phosphorylation. Genes Dev..

[44] Lin M., Zhao Z., Yang Z., Meng Q., Tan P., Xie W., Qin Y., Wang R.F., Cui J. (2016). USP38 inhibits type I interferon signaling by editing TBK1 ubiquitination through NLRP4 signalosome. Mol Cell.

[45] Cui J., Li Y., Zhu L., Liu D., Songyang Z., Wang H.Y., Wang R.F. (2012). NLRP4 negatively regulates type I interferon signaling by targeting the kinase TBK1 for degradation via the ubiquitin ligase DTX4. Nat. Immunol..

[46] Borden E.C., Sen G.C., Uze G., Silverman R.H., Ransohoff R.M., Foster G.R., Stark G.R. (2007). Interferons at age 50: past, current and future impact on biomedicine. Nat. Rev. Drug Discov..

[47] Xu Y., Ding J., Wu L.Y., Chou K.C. (2013). iSNO-PseAAC: predict cysteine S-nitrosylation sites in proteins by incorporating position specific amino acid propensity into pseudo amino acid composition. PLoS One.

[48] Kotas M.E., Medzhitov R. (2015). Homeostasis, inflammation, and disease susceptibility. Cell.

[49] Jia M., Qin D., Zhao C., Chai L., Yu Z., Wang W., Tong L., Lv L., Wang Y., Rehwinkel J., Yu J., Zhao W. (2020). Redox homeostasis maintained by GPX4 facilitates STING activation. Nat. Immunol..

[50] Zong Z., Zhang Z., Wu L., Zhang L., Zhou F. (2021). The functional deubiquitinating enzymes in control of innate antiviral immunity. Adv. Sci..

[51] Ribet D., Cossart P. (2010). Pathogen-mediated posttranslational modifications: a re-emerging field. Cell.

[52] Barnett S.D., Buxton I.L.O. (2017). The role of S-nitrosoglutathione reductase (GSNOR) in human disease and therapy. Crit. Rev. Biochem. Mol. Biol..

[53] Wu K.Y., Zhang Y.Y., Su W.T., Chen C. (2013). GSNOR: a novel regulator of inflammation. Prog. Biochem. Biophys..

[54] Xiang W., Zhang Q., Lin X., Wu S., Zhou Y., Meng F., Fan Y., Shen T., Xiao M., Xia Z., Zou J., Feng X.H., Xu P. (2016). PPM1A silences cytosolic RNA sensing and antiviral defense through direct dephosphorylation of MAVS and TBK1. Sci Adv.

[55] Li X., Yang M., Yu Z., Tang S., Wang L., Cao X., Chen T. (2017). The tyrosine kinase Src promotes phosphorylation of the kinase TBK1 to facilitate type I interferon production after viral infection. Sci. Signal..

[56] Tu D., Zhu Z., Zhou A.Y., Yun C.H., Lee K.E., Toms A.V., Li Y., Dunn G.P., Chan E., Thai T., Yang S., Ficarro S.B., Marto J.A., Jeon H., Hahn W.C., Barbie D.A., Eck M.J. (2013). Structure and ubiquitination-dependent activation of TANK-binding kinase 1. Cell Rep..

[57] Saul V.V., Niedenthal R., Pich A., Weber F., Schmitz M.L. (2015). SUMO modification of TBK1 at the adaptor-binding C-terminal coiled-coil domain contributes to its antiviral activity. Biochim. Biophys. Acta.

[58] Li X., Zhang Q., Ding Y., Liu Y., Zhao D., Zhao K., Shen Q., Liu X., Zhu X., Li N., Cheng Z., Fan G., Wang Q., Cao X. (2016). Methyltransferase Dnmt3a upregulates HDAC9 to deacetylate the kinase TBK1 for activation of antiviral innate immunity. Nat. Immunol..

[59] Yamada T., Horimoto H., Kameyama T., Hayakawa S., Yamato H., Dazai M., Takada A., Kida H., Bott D., Zhou A.C., Hutin D., Watts T.H., Asaka M., Matthews J., Takaoka A. (2016). Constitutive aryl hydrocarbon receptor signaling constrains type I interferon-mediated antiviral innate defense. Nat. Immunol..

[60] Oka Y., Hamada M., Nakazawa Y., Muramatsu H., Okuno Y., Higasa K., Shimada M., Takeshima H., Hanada K., Hirano T., Kawakita T., Sakaguchi H., Ichimura T., Ozono S., Yuge K., Watanabe Y., Kotani Y., Yamane M., Kasugai Y., Tanaka M., Suganami T., Nakada S., Mitsutake N., Hara Y., Kato K., Mizuno S., Miyake N., Kawai Y., Tokunaga K., Nagasaki M., Kito S., Isoyama K., Onodera M., Kaneko H., Matsumoto N., Matsuda F., Matsuo K., Takahashi Y., Mashimo T., Kojima S., Ogi T. (2020). Digenic mutations in ALDH2 and ADH5 impair formaldehyde clearance and cause a multisystem disorder, AMeD syndrome. Sci Adv.

[61] Li Y., Zhang Y., Wang L., Wang P., Xue Y., Li X., Qiao X., Zhang X., Xu T., Liu G., Li P., Chen C. (2017). Autophagy impairment mediated by S-nitrosation of ATG4B leads to neurotoxicity in response to hyperglycemia. Autophagy.

