# Supplementary material for: GSNOR facilitates antiviral innate immunity by restricting TBK1 cysteine S-nitrosation
Source: Redox Biol. 2021 Oct 18;47:102172. doi: 10.1016/j.redox.2021.102172 (PMC8577438; doi:10.1016/j.redox.2021.102172)
Supplement: Multimedia component 1 [file mmc1.docx]

**Supplemental material**

**Supplementary Fig. 1** ***Gsnor* deficiency impairs type I interferon production**  *Ifnb-1*, *Ifna4*, *Mx1* and *Ifit1* mRNA expression levels in WT and *Gsnor* KO L929 cells transfected with RNA virus mimics poly (I:C) **(A)** and DNA virus mimic HSV-60 **(B)** for the indicated times. All data were representative of three independent experiments with similar results. The mRNA level of each gene was quantified by using quantitative real-time PCR, with a normalization to *β-actin*. Data are mean ± SD. ** *p* < 0.01, *** *p* < 0.001, **** *p* < 0.0001, two-tailed unpaired Student’s *t*-test.

**Supplementary Fig. 2** **GSNOR knockdown inhibited virus-induced phosphorylation of TBK1 and IRF3**

HaCaT cells were transfected with control siRNA (siRNA NC) and siRNA for *GSNOR* (siGSNOR) for 12 h, then were infected with HSV-1 (MOI = 1) or SeV (20 HAU/mL) for the indicated times. Immunoblot of lysates of the harvested cells showing that GSNOR knockdown reduced the HSV-1 (**A**) and SeV (**B**) virus-induced phosphorylation of IRF3 and TBK1.

**Supplementary Fig. 3** **Overexpression of GSNOR potentiated virus-induced phosphorylation of TBK1 and IRF3 levels**

Overexpression of GSNOR potentiates HSV-1 **(A)** and SeV **(B)** virus-induced phosphorylation of TBK1 and IRF3 levels in HaCat cells compared to those transfected with the empty vector (Vector).

**Table S1.** Primer pairs for detecting mRNA expression levels of target genes

| **Primer** | **Sequence (5’-3’)** |
| --- | --- |
| *Ifnb1* Forward | CAGCTCCAAGAAAGGACGAAC |
| *Ifnb1* Reverse | GGCAGTGTAACTCTTCTGCAT |
| *Ifna4* Forward | TGATGAGCTACTACTGGTCAGC |
| *Ifna4* Reverse | GATCTCTTAGCACAAGGATGGC |
| *Mx1* Forward | GACCATAGGGGTCTTGACCAA |
| *Mx1* Reverse | AGACTTGCTCTTTCTGAAAAGCC |
| *Ifit1* Forward | CTGAGATGTCACTTCACATGGAA |
| *Ifit1* Reverse | GTGCATCCCCAATGGGTTCT |
| HSV-1 *UL30* Forward | CATCACCGACCCGGAGAGGGAC |
| HSV-1 *UL30* Reverse | GGGCCAGGCGCTTGTTGGTGTA |
| *Actin* Forward | GATGGTGGGAATGGGTCAGA |
| *Actin* Reverse | TCCATGTCGTCCCAGTTGGT |
